# Can they touch? A novel mental motor imagery task for the assessment of back pain

**DOI:** 10.3389/fpain.2023.1189695

**Published:** 2024-02-05

**Authors:** H. Branch Coslett, Jared Medina, Daria Kliot Goodman, Yuchao Wang, Adam Burkey

**Affiliations:** ^1^ Department of Neurology, Hospital of the University of Pennsylvania, Philadelphia, PA, United States; ^2^ Department of Psychology, University of Delaware, Newark, DE, United States; ^3^ Anesis Spine and Pain Care, Renton, WA, United States

**Keywords:** motor imagery, low back pain, pain measurement, body schema, motor cognition

## Abstract

**Introduction:**

As motor imagery is informed by the anticipated sensory consequences of action, including pain, we reasoned that motor imagery could provide a useful indicator of chronic back pain. We tested the hypothesis that mental motor imagery regarding body movements can provide a reliable assessment of low back pain.

**Methods:**

Eighty-five subjects with back pain and forty-five age-matched controls were shown two names of body parts and asked to indicate if they could imagine moving so that the named body parts touched. Three types of imagined movements were interrogated: movements of arms, movements of legs and movements requiring flexion and/or rotation of the low back.

**Results:**

Accuracy and reaction times were measured. Subjects with back pain were less likely to indicate that they could touch body parts than age-matched controls. The effect was observed only for those movements that required movement of the low back or legs, suggesting that the effect was not attributable to task difficulty or non-specific effects. There was an effect of pain severity. Compared to subjects with mild pain, subjects with severe pain were significantly less likely to indicate that they could move so that named body parts touched. There was a correlation between pain ratings and impaired performance for stimuli that involved the lower but not upper body.

**Discussion:**

As the Can They Touch task is quick, easy to administer and does not require an explicit judgment of pain severity, it may provide useful information to supplement the assessment of subjects with chronic pain.

## 1 Introduction

Pain is embodied—that is, pain is a noxious sensation that involves specific body components such as the back, head, or arm. As pain is an internal state, its properties and severity are inherently subjective and elusive. The psychometric properties of pain are often assessed using questionnaires that measure the impact of pain on daily activities such as sleep and walking [(1–3) see (4) for review]. The reliability of such self-reported pain measures is debated (5, 6).

Measures that interrogate the sensory-motor processes that induce pain may provide an additional appraisal of pain severity (7). We suggest that mental motor imagery may provide a useful indicator of pain. The “simulation” or “functional equivalence” model proposes that mental motor imagery shares internal representations with overt motor execution (8, 9). Although recent work suggests that cognitive factors influence mental motor imagery [e.g., (10)], multiple lines of evidence support the claim that motor imagery and action are similar in many respects. Behaviorally, it has been observed that the time required to imagine performing a task is highly correlated with the time needed to execute the task (11–13). Imagined movements also show speed-accuracy tradeoffs as in real movements (8, 13, 14). Mental simulation of action can also attenuate sensory feedback (15) as well as elicit autonomic responses qualitatively similar to those observed with action [e.g., (16), see (17, 18) for review]. Finally, functional neuroimaging data have revealed increased activity in similar (but not identical) somatotopically-matched brain regions during mental motor imagery and motor execution (19–21; cf. 22).

If action and mental motor imagery are subserved by many of the same brain networks and computations, factors that influence real movements like current body configuration (12) and pain should similarly impact motor imagery performance. Such effects have been demonstrated. Parsons demonstrated that when subjects are asked to judge if a stimulus depicts a right or left hand, reaction times are shorter if the stimulus is in the same position as their unseen hand (11, 23, 24). Using a similar task, we showed that localized pain in one upper extremity leads to slower reaction times in judgments involving the painful as compared to the unaffected arm (25). This reaction time difference was eliminated by a treatment that reduced pain (26). As recently noted in a meta-analysis, mental motor imagery tasks involving left-right discrimination (27) have demonstrated effects of pain on mental motor imagery for the hand (28, 29), leg (30, 31), face (32), and low back (33). To date, however, evidence from left-right discrimination tasks is less clear for studies investigating subjects with chronic pain in the neck and low back, with some studies showing no effect (34).

We report an investigation of the utility of a novel explicit motor imagery task in the assessment of subjects with low back or low back and leg pain. We predicted that subjects with low back pain would be slower to respond or less likely than controls to respond in the affirmative when asked if they could touch their body parts if the actual movement would involve the painful body part.

## 2 Materials and methods

### 2.1 Subjects

Three groups of subjects were included. There were 35 subjects with pain involving only their lower back (LBP) of at least 3 months duration (mean age 57 ± 13 years; 19 females). In addition, there were 46 subjects with lower back pain of at least 3 months duration that extended into one or both legs (LBLP, mean age 53 ± 10 years, 22 females). Finally, there were 45 asymptomatic control subjects with no history of chronic pain (AC, 54 ± 14 years; 24 females). LBP and LBLP groups did not differ significantly from AC subjects with respect to age or gender. Subjects with back pain and most asymptomatic subjects were recruited from the Pain Control Center at the University of Pennsylvania; approximately one third of asymptomatic subjects were recruited through advertisements approved by the Institutional Review Board. The sample size was not predetermined; we recruited all willing participants who met inclusion and exclusion criteria until recruitment efforts were discontinued because of the departure of research staff. Subjects with pain were tested at the time of a regularly scheduled visit to the Pain Control Center while taking their usual medications over an approximately 2-year interval. Subjects rated their pain severity in two ways. First, subjects were asked to indicate the severity of their pain at the time of testing. Second, subjects were asked to rate their typical pain severity during movement. Subjects rated their pain with a 0–10 visual analog scale. Additionally, subjects completed the McGill Short Form Pain questionnaire and the Visual Analog Mood Scale. Mean ratings for pain at the time of testing were 6.03 ± 3.12 and 6.11 ± 2.64 for the low back pain and low back plus leg pain groups, respectively; mean pain ratings for Pain with movement were 7.24 ± 2.86 and 7.35 ± 2.86 for the low back pain and low back plus leg pain groups, respectively; groups did not differ in pain ratings (*p *= .90 and .86 respectively).

Subjects were paid for their participation. Consent was obtained according to the Declaration of Helsinki; the project was approved by the University of Pennsylvania IRB.

### 2.2 Stimuli

Stimuli included 60 pairs of body part names; there were 20 trials in each of the following conditions: Upper Body trials in which the named body parts were located on the head, arms or torso above the waist (e.g., “left wrist—nose”); Lower Body trials on which the body parts were both located below the waist (e.g., “left heel—right knee”); and Across Body trials on which one body part was above and the other below the waist (e.g., “left hand—right foot”). Half of the pairs in each of the 3 conditions were expected to generate a “No” response, either because they were physically impossible (e.g., “left knee—left shin”) or because they were expected to require unusual flexibility (e.g., “right ear—left shin”).

Although the stimuli were initially selected on the basis of greater than 90% agreement in a cohort of young healthy subjects, when employed with the subjects described below there was greater variability in performance. The analyses reported below were based on 43 items for which asymptomatic subjects exhibited greater than 85% agreement. We note that an analysis was also performed on the 23 items for which there was >92% agreement in controls; the results were very similar to those reported here. Furthermore, as will be demonstrated below, the same pattern of results is observed if one restricts the analysis to items to which control subjects answered “yes”. Thus, varying the composition of the items in the task does not substantially alter the basic findings.

Finally, we performed a measure of item intercorrelation [Cronbach alpha analysis, (35)] in order to determine if the 43 items with greater than 85% agreement were internally consistent. The Cronbach alpha was .709 for the Lower Body, .684 for the Across Body and .814 for the Upper Body stimuli. Alpha values 0.60 and below indicate a lack of consistency among the items, and values above 0.90 indicate redundancy among the items (36).

### 2.3 Task

**Figure 1:**
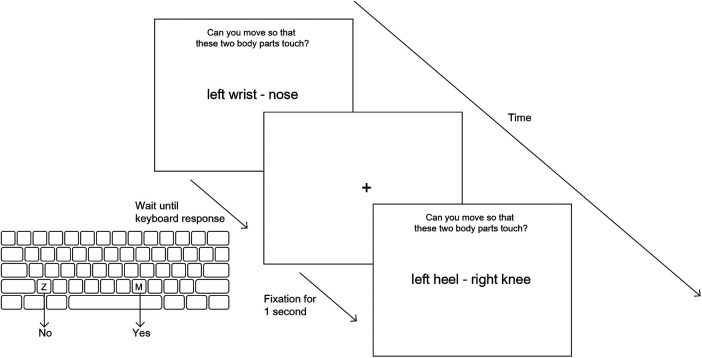
Overview of Can they touch task. Each stimulus (a pair of body parts) is displayed on screen until the subject makes a keyboard response (“z” for no and “m” for yes) with their left or right index finger. A fixation cross is shown for 1 s before the next stimulus screen.

Subjects sat in a comfortable chair facing a computer screen. They were told that they would see two names of body parts and were requested to indicate if they would be able to move so that the named body parts could touch (see Figure 1). Subjects were instructed not to move and were observed by study personnel during the testing. Practice trials were not provided but, consistent with our practice in other studies involving mental motor imagery, the task was discussed in detail before testing commenced. In particular, it was emphasized that subjects were not to move but to imagine moving while performing the task. Additionally, it was emphasized that that there was not a “correct” answer and that their response should be based upon their assessment of their capacity at the time of testing. Finally, a number of examples were discussed by research personnel and the subject to be sure that subjects understood the task.

Subjects maintained their hands in the palm down position with the index fingers of the left and right hands over the “z” and “m” keys, respectively; they depressed the “z” key for “no” and the “m” key for “yes”. Subjects were instructed to respond quickly, but accurately. Although formal ratings of pain were not collected after the task, subjects were asked if their pain was altered by the testing; no subject reported noticing an increase in pain during or after the test.

Stimuli were presented in a different random sequence with each administration of the task. Each trial began with the presentation of a fixation cross that persisted for one second before being replaced the two body part names. The trial was terminated by depressing the “z” or “m” key. A new fixation cross was presented one second after the subject's response. On average, the task lasted approximately 7–10 min. The task was developed by study personnel using E-Prime software.

### 2.4 Statistical analysis

Two-way ANOVAs with a within-subject variable (Condition: Upper Body, Across Body, Lower Body) and a between-subject variable (either Group: LBP, LBLP, AC, or Pain Severity: Mild, Moderate, Severe) were performed separately for Agreement and reaction time (RT) data. When assumptions of sphericity were violated, Greenhouse-Geisser corrections were used. *Post hoc* pairwise comparisons were performed as warranted by ANOVA results. The same analyses were repeated excluding stimuli that were physically impossible (50% of all stimuli) to rule out the potential concern that these trials did not involve mental motor imagery.

Linear correlations were tested between our outcome variables (Agreement and RT) and pain ratings (Present Pain and Pain with movement) for subjects with pain (LBP, LBLP) within each Condition (Upper Body, Across Body, Lower Body). This is to further examine the validity of our outcome variables as a potential alternative measure for pain that is specific to pain location.

Furthermore, positive and negative predictive values (i.e., true positives/negatives over total positives/negatives, respectively) were calculated to explore the clinical value of using Can They Touch test as a supplementary measure of pain.

## 3 Results

All data and stimuli can be found online at https://osf.io/wv3xc/. Reaction time (RT) and yes/no responses were recorded for each trial. Consistency of response was assessed for each item. Investigator's observations of the behavior of test-takers suggested that some participants performed poorly because of factors such as a failure to engage in the task, an inability to understand the task, or an inability to stay on task. In an effort to identify those subjects, six items were identified on which asymptomatic subjects made no errors (e.g., “right thumb—left thumb” and “left shin—left knee”). Subjects who made 3 or more errors on these 6 items were omitted from the analysis. On this basis, LBP and 2 LBLP subjects were omitted from analysis.

For many items, there is no “correct” response. For example, whereas the vast majority of individuals would be unable to touch their left heel to their right shoulder, exceptionally lithe, flexible people may be able to do so. In light of this consideration, we were unable to determine a percent correct score; instead, we employed an Agreement score reflecting the proportion of trials on which the subject's response was in accord with the consensus (defined as >85%) of controls. Only responses on which the subject agreed with the consensus as defined above were included in the RT analyses. The average agreement scores (with standard deviations) for AC, LBP, LBLP groups were 92 ± 5%, 85 ± 11%, and 85 ± 11%, respectively.

### 3.1 Group analyses

**Figure 2:**
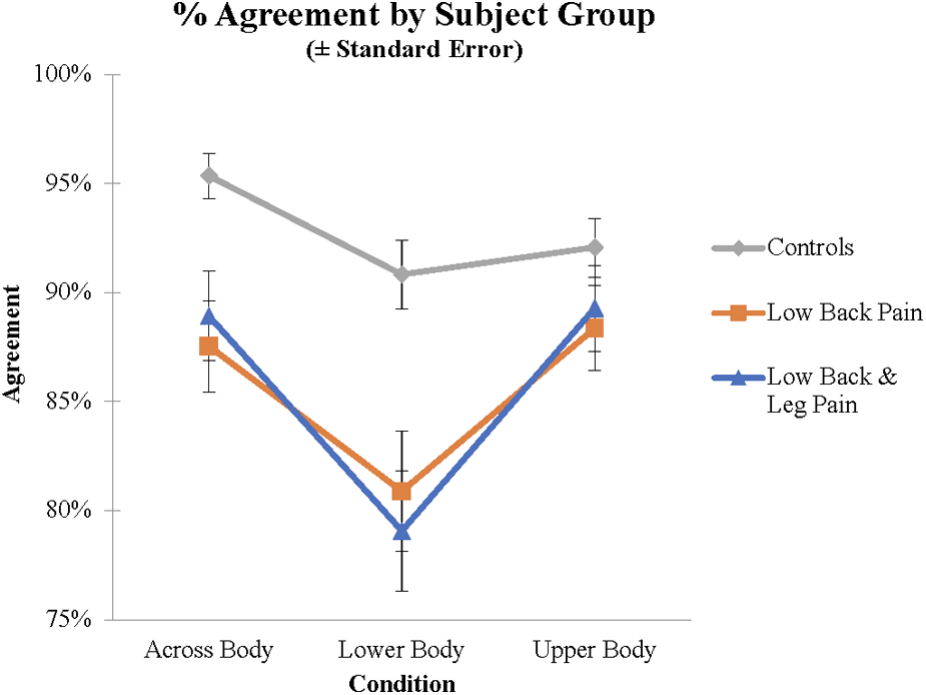
Agreement scores for the different groups and different stimuli demonstrating the group by stimulus interaction.

First, ANOVAs were performed for the RT and Agreement data with group (LBP, LBLP, AC) as a between-subject variable and Condition (Upper Body, Across Body, Lower Body) as a within-subject variable. For the Agreement analysis there were main effects of Condition (*F*(1.84, 226.25) = 19.1, *p *< .001, *η*^2^ = 0.134) and Group (*F*(2, 123) = 8.48, *p *< .001, *η_p_*^2^ = 0.121). As indicated in Table 1, the proportion of trials on which subjects produced the normative response as defined by “Agreement” criteria above, was highest in the Across Body and Upper Body and lowest for the Lower Body stimuli. *Post hoc* tests demonstrated that the yes rate for Lower Body stimuli was significantly lower than that for Across Body or Upper Body (both *p *< .001); Upper Body and Across Body did not differ (*p *= .521). There was also a significant effect of Group; AC subjects (92.8%) were more likely to say yes than either LBP (85.6%) or LBLP (85.8%) subjects (both *p *< .001); LBP and LBLP subjects did not differ (*p *= .936). There was also an interaction between group and condition (*F*(3.68, 226.25) = 2.58, *p *= .043, *η*^2^ = 0.040; see Figure 2). *Post hoc* tests demonstrated that the difference between Lower Body and Upper Body stimuli was significantly greater for both groups of back pain subjects than for controls (*p *= .004 for LBLP and *p *= .040 for LBP). Finally, there was a significantly greater decrement in performance for the Lower Body, compared to Across Body stimuli, for the LBLP relative to ACs (*p *= .033); the difference between LBP and ACs was not significant (*p *= .247).

**Table 1:** Percent agreement as a function of group and stimulus type (±standard error).

	Upper body	Across body	Lower body	All stimuli
Controls	92.1 ± 1.4	95.4 ± 1.0	90.8 ± 1.6	92.5 ± 1.0
Lower back pain	88.4 ± 1.9	87.5 ± 2.1	80.9 ± 2.8	85.2 ± 1.7
Lower back/Leg pain	89.3 ± 2.0	88.9 ± 2.1	79.1 ± 2.8	85.2 ± 1.7
Total	90.0 ± 0.9	90.8 ± 1.0	83.8 ± 1.4	87.8 ± 0.9

Analysis of RT data demonstrated a significant effect of Condition (*F*(2, 246) = 39.7, *p *< .001, *η*^2^ = 0.244; see Table 2). RTs for Lower Body stimuli (5,822.8 ms) were significantly slower than either Across Body (5,022.6 ms) or Upper Body (4,780.1 ms) stimuli (*p *< .001 for both). Upper Body responses were significantly faster than Across Body responses (*p *= .049). There was a trend towards a main effect of Group (*F*(2, 123) = 2.618, *p *< .077), *η*^2^ = 0.041; AC (4,708.4 ms) were significantly faster than LBLP subjects (5,570.8 ms; *p *= .028), but did not differ from LBP subjects (5,346.2 ms; *p *= .129); LBP and LBLP subjects did not differ (*p *= .589). There was no interaction between group and condition (*F*(4, 246) = .775, *p *= .542, *η*^2^ = 0.012).

**Table 2:** Reaction times as a function of group and stimulus type (ms, ±standard error).

	Upper body	Across body	Lower body	All stimuli
Controls	4,297.4 ± 186.1	4,629.2 ± 205.7	5,198.7 ± 267.4	4,708.4 ± 209.3
Lower back pain	5,004.9 ± 363.1	5,056.3 ± 300.4	5,977.4 ± 388.9	5,346.2 ± 332.1
Lower back/Leg pain	5,037.8 ± 281.6	5,382.5 ± 390.1	6,292.3 ± 365.3	5,570.8 ± 312.9
Total	4,780.1 ± 160.3	5,022.6 ± 181.6	5,822.8 ± 199.4	5,521.7 ± 166.7

In summary, LBP and LBLP subjects were significantly less likely than ACs to indicate that they could move so that named body parts could touch. Importantly, this difference was greatest for the Lower Body stimuli. RT analyses revealed no interaction between group and condition.

### 3.2 Effect of pain severity

**Figure 3:**
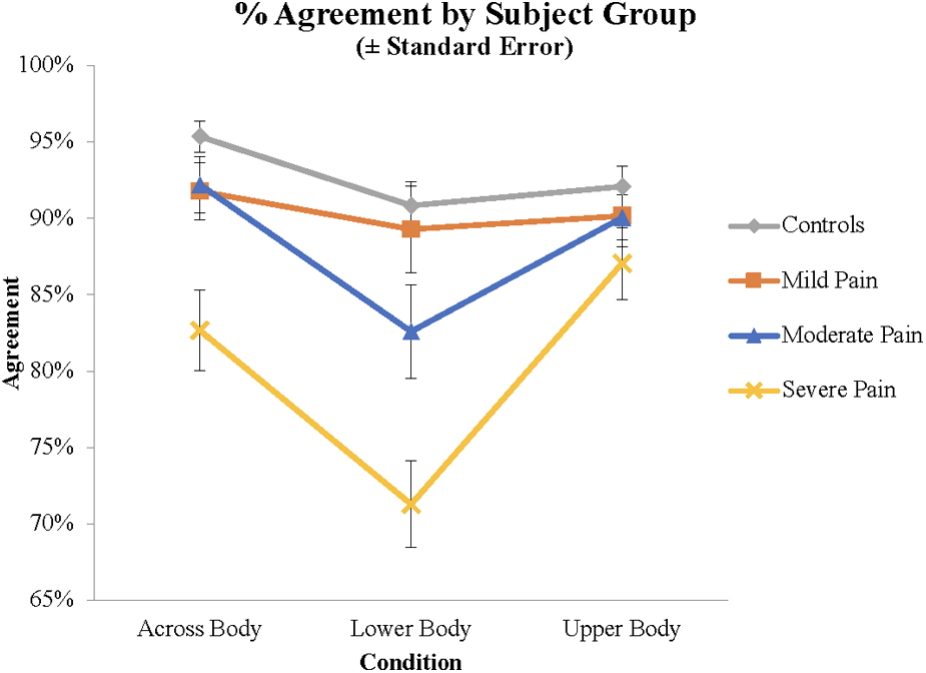
Agreement scores demonstrating the interaction between pain severity and stimulus type.

As there were no significant differences between the LBP and LBLP groups, these groups were combined in subsequent analyses.^1^ Factors for these ANOVAs included Pain Severity at the time of testing (Mild, Moderate, Severe) and Condition (Upper Body, Across Body, Lower Body). Pain ratings from 1 to 4 were categorized as Mild (*N* = 21), 5–7 as Moderate (*N* = 28), and 8–10 as Severe (*N* = 32). Analysis of the Agreement data demonstrated a main effect of Present Pain Severity (*F*(2, 78) = 8.42, *p *< .001, *η*^2^ = 0.178). As indicated in Figure 3, subjects with mild, moderate and severe pain responded in the anticipated fashion on 90.4%, 88.2% and 80.3% of trials, respectively. Pair-wise comparisons demonstrated that subjects with Severe pain were less likely to respond in the normative manner than Moderate (*p *= .002) and Mild (*p *< .001) pain groups. There was also a main effect of Condition (*F*(1.84, 143.21) = 15.6, *p *< .001, *η*^2^ = 0.166); subjects responded in the normative fashion for 89.1%, 88.9.% and 81.1% of trials on the Upper Body, Across Body and Lower Body items, respectively. Pair-wise comparisons demonstrated that Lower Body items differed from both other conditions (*p *< .001), but Upper Body and Across Body items did not differ (*p *= .890).

Finally, there was a Condition by Group interaction (*F*(3.75, 143.2) = 3.75, *p *= .008, *η*^2^ = 0.088). Subjects with Severe Present Pain exhibited a significantly greater decrement on the Lower Body compared to Upper Body stimuli than subjects with Mild Present Pain (*p *= .003), with a trend for subjects with Moderate Present Pain (*p *= .063). Similarly, for the comparison between Across Body and Upper Body stimuli, subjects with Severe Present Pain exhibited a greater deficit than subjects with Moderate Present Pain (*p *= .023) and Mild Present Pain. Finally, for the comparison between Lower and Across Body stimuli, subjects with Severe Present Pain exhibited a significantly greater decrement for Lower Body than Across Body stimuli than subjects with Mild (*p *= .017) or Moderate Present Pain (*p *= .048).

ANOVA performed on the RT data demonstrated main effects of Condition (*F*(2, 156) = 24.8, *p *< .001, *η*^2^ = 0.242). However, the main effect of Severity (*F*(2, 78) = .27, *p *= .766, *η*^2^ = 0.007) and the Condition by Group interaction were not significant (*F*(4, 156) = 1.16, *p *= .332, *η_p_*^2^ = 0.029).

In summary, there was a significant effect of Present Pain severity. Subjects with Severe Present Pain were significantly less likely to indicate that they could move so that named body parts could touch than all other groups of subjects. Once again, there was a group by condition interaction demonstrating that subjects with severe pain were disproportionately impaired on the Lower Body and Across Body stimuli.

### 3.3 Analysis of “Yes” responses

Determining if two named body parts can be made to touch is assumed to require a mental simulation of the action that would be required to bring the body parts together. In order to avoid response bias, the stimuli were constructed such that the anticipated answer was “no” in 50% of trials. For some of these stimulus pairs, however, it is physically impossible to make the body parts touch (e.g., “left shin—left knee”). Mental motor imagery may not have been needed on these trials In order to provide a stronger test of the motor imagery hypothesis, we performed an analysis in which only trials for which the anticipated response was “yes” were included. One subject was eliminated from previous analyses, due to poor performance on “yes” trials. Severity was a between subject factor and Condition was a within subject factor. For the Agreement analysis there were significant main effects of Severity (*F*(3, 121) = 13.44, *p *< .0001, *η*^2^ = 0.250), and Condition (*F*(2, 242) = 13.96, *p *< .0001, *η*^2^ = 0.103). Pairwise comparisons revealed that Severe pain subjects (75.2%) were less likely than Mild pain subjects (89.5%) and Moderate pain subjects (87.9%; all *p*s < .001) to respond in the normative fashion. The other groups did not differ. Pain subjects were more likely to respond like controls in the Upper Body trials (89.8%) and Across Body trials (88.1%) than Lower Body trials (81.6%; both *p *> .001), but Upper and Across Body trials did not differ. There was also a significant interaction between Severity and Condition (*F*(6, 242) = 5.34, *p *< .001, *η*^2^ = 0.117).

RT analyses demonstrated significant effects of Severity (*F*(1, 121) = 4.149, *p *< .008, *η*^2^ = 0.033) and Stimulus (*F*(2, 242) = 33.999, *p *< .001, *η_p_*^2^ = 0.219) but no interaction (*F*(6, 242) = .682, *p *= .664, *η*^2^ = 0.017).

In summary, when the analysis is restricted to trials on which the modal response is “yes”, there are significant main effects of pain severity and stimulus type and most importantly, an interaction demonstrating that subjects with severe pain are disproportionately impaired on trials on which they must imagine moving their painful back and legs.

### 3.4 Correlations

For subjects with back pain (LBP and LBLP), correlations were performed between Present Pain and Pain with movement ratings and Agreement scores for the Upper Body, Across Body, and Lower Body stimuli. There was a highly significant inverse correlation between Present pain rating for the Lower Body (*r*(75) = −.407, *p *< .001) and a trend for a significant inverse correlation between pain and Across Body scores (*r*(75) = −.213, *p *= .061). The correlation for Upper Body items was not significant (*r*(75) = −.077, *p *= .500). Similar analyses for Pain with movement scores yielded no significant correlations (Lower Body: *r*(75) = −.170, *p *= .138; Across Body: *r*(75) = −.179, *p *= .118; Upper Body: *r*(75) = −.059, *p *= .606). There were no significant correlations for RT and pain ratings.

As there was a significant correlation between Pain with movement and Present pain ratings (*r*(79) = .389, *p *< .001), a partial correlation between Present pain ratings and Agreement scores controlling for Pain with movement was performed. We found a significant inverse correlation between Lower Body performance and pain score (*r*(75) = −.389, *p *= .028). The partial correlations for Across Body and Upper Body were not significant (*r*(75) = −.079, *p *= .669 and *r*(75) = .081, *p *= .661 respectively).

### 3.5 Discriminating subjects with pain from controls

Previous investigations have established that motor imagery tasks demonstrate that groups of subjects with pain differ from controls (27, 28, 30). In the clinical setting, however, one evaluates and treats individuals rather than groups. A task that reliably discriminates between individuals with and without pain that did not depend on verbal ratings would be of potential clinical relevance. We addressed the ability of the Can They Touch task to discriminate individual subjects with back pain from asymptomatic subjects by determining the Sensitivity, Specificity, Positive Predictive Value and Negative Predictive Value of the task. To this end, we calculated the normal range of performance as the control mean ± 2 SDs and determined the proportion of each group of subjects with Mild, Moderate and Severe pain who fell outside the normal range (see Table 3). We found that 0/21 subjects (0%) with Mild, 6/28 subjects (21.4%) with Moderate and 16/32 subjects (50%) with Severe pain fell outside the normal range. The Sensitivity, Specificity as well as Positive and Negative Predictive Values for subjects with different pain severities are presented in Table 4.

**Table 3:** Percent of subjects exhibiting abnormal performance.

	Across body	Lower body	Upper body	Total score
Control	2.20%	4.50%	8.90%	2.20% (1/45)
Mild Pain	4.80%	14.30%	4.80%	0.0% (0/21)
Moderate Pain	17.90%	25.00%	3.60%	21.4% (6/28)
Severe Pain	34.40%	43.80%	15.60%	50.0% (16/32)

**Table 4:** Sensitivity, specificity and predictive value.

	All pain	Mild pain	Moderate pain	Severe pain
Sensitivity	0.271	0	0.214	0.5
Specificity	0.978	0.978	0.978	0.977
Positive Pred. Value	0.957	0	0.857	0.941
Negative Pred. Value	0.427	0.677	0.666	0.733

## 4 Discussion

We report data from a novel mental motor imagery task in which subjects were asked to make explicit judgments about their ability to move so that two body parts could be made to touch. As predicted, we found that subjects with low back pain were less likely than controls to indicate that they would be able to move such that named body parts could touch. This effect is not simply attributable to the novelty of the task or non-specific factors such as medication effects or arousal, as the difference between asymptomatic and pain subjects was significant for those items involving the lower back and legs but not the upper body. Furthermore, there was a significant effect of pain severity in that the difference between pain subjects and controls was greatest for subjects with severe back pain. As the task requires mental motor imagery and interrogation of the body schema, we believe that the Can Touch task primarily interrogates cognitive aspects of pain.

We believe these findings are important for a number of reasons. First, we report a novel task that not only provides a measure of low back/leg pain but also, unlike previous tasks [e.g., (33)], demonstrates an effect of the severity of back pain on performance. Second, as we (28, 30) have argued previously, mental motor imagery tasks may complement questionnaire-based assessments of physical functioning. Motor imagery tasks avoid the recall bias inherent in querying past ability to perform specific motor tasks. Additionally, the covert nature of the test as an indicator of pain may also reduce measurement bias.

Third, unlike previous motor imagery tasks that have demonstrated significant differences between groups of subjects (25, 26, 28, 33, 37, 38), our task provides information relevant to the individual subject rather than just the group. As clinical practice entails decisions regarding the diagnosis and treatment of the individual subject, this is an important distinction. Unlike all previous mental motor imagery tasks of which we are aware, the task reported here not only demonstrates significant group effects but, as indicated in Table 4, exhibits excellent specificity and good Positive and Negative Predictive values. A limitation of the task in its current form, however, is low sensitivity.

A number of potential explanations for the low sensitivity may be identified. As explicit motor imagery has been shown to cause pain and swelling in subjects with chronic pain (29, 39, 40), it is possible that some subjects do not follow task instructions. For example, if subjects found that imagined movement of their body was associated with pain, they might choose to answer the questions on the basis of what another person might be able to do. A second possible reason lies in the different stages of central sensitization that the subjects may be undergoing. Central sensitization describes the transition from nociceptive to nociplastic pain due to altered central nervous processing (41), and is hypothesized to occur as acute pain evolves into chronic pain (42–44). Although all our subjects had experienced pain for longer than 3 months, there was a substantial range with respect to the duration of pain; it is possible that some of our subjects were at earlier stages of central sensitization, which might not have impacted their motor imagery performance considerably. Future studies should consider administering the Central Sensitization Inventory [CSI, (45)] to investigate if CSI scores serve as an additional predictor of pain severity.

Although the Can They Touch task is relatively quick and easy to administer, it is clearly more time and resource intensive than traditional pain ratings. At least at this stage in its development, we suggest that the Can They Touch task may prove useful as an indicator of change over time or response to therapy. As subjects are asked to make judgments regarding their ability to touch body parts at the time of testing, one might expect that a change in pain status would be reflected in task performance. Although the data are quite limited, we note that two subjects were tested before and after treatment for back pain with local injections. After treatment, both subjects were more likely to respond like controls on items involving the lower body. The hypothesis that our mental motor imagery task is reliant upon an on-line, constantly updated representation of the body that would change in response to activity or therapy was supported by our previous demonstration that the asymmetry on a right/left hand discrimination task in subjects with Complex Regional Pain Syndrome was eliminated after therapy (26). The potential utility of the task as a measure of change over time requires additional research.

Performance on Left Right Judgment Tasks (LRJT) such as the hand laterality task have, with few exceptions, been found to assess appendicular pain but have proven less useful in the assessment of trunk pain (34, 46, 47) [see meta-analysis in (27)]. This difference was attributed by Breckenridge et al. (27) to different cortical representations of axial and appendicular pain. Limbs primarily serve to interact with the external world, which requires more conscious control, whereas the trunk mainly maintains balance and posture without conscious effort. Neurophysiologically, while both interoceptive and exteroceptive signals from limbs and the trunk may be integrated along the posterior-anterior axis of the insula to generate a bodily awareness [see review in (48)], exteroceptive sensory inputs from the limbs are represented in sensory cortex and are implicated in sensorimotor coordination and motor execution. Behaviorally, though virtually all subjects can suppress overt execution during motor imagery involving the extremities, this may be more difficult for motor imagery involving the trunk [see review by (49)]. Subjects may have greater access and control of their limbs as compared to the trunk, leading to easier selective engagement of motor imagery of the limbs rather than the trunk in LRJT. The Can They Touch task may overcome this limitation as subjects did not explicitly engage motor imagery of the trunk to determine, for example, if the right hand could be made to touch the left ankle. Thus, our task differed from many motor imagery tasks involving the trunk in that simulated actions involved movement of the entire body rather than the trunk alone.

One interesting aspect of our findings is that Present pain ratings, but not Pain with action ratings, correlated with performance on the task. This is consistent with the results of Moseley et al. (40) but contrasts with previous reports from our laboratory in which performance on mental motor imagery tasks involving the hand (28) and foot (30) correlated with Pain with movement but not Present pain ratings. One potential account of this discrepancy appeals to the distinction between implicit and explicit motor imagery. In the task described here, subjects are encouraged to employ an explicit mental rotation strategy and, in informal debriefing, consistently reported doing so. Similarly, Moseley et al. (40) specifically instructed subjects to imagine making movements of the arm. In contrast, in hand and foot laterality tasks, subjects are simply asked to judge whether a stimulus is a right or left hand or foot; although abundant evidence [see review in (27)] suggests that subjects perform the task by mentally rotating their body to match the stimulus, in our experience most subjects do not report explicitly rotating their extremity. One potential account of the fact that explicit motor imagery correlates with ratings of state pain, whereas implicit motor imagery correlates with ratings of Pain with movement, is that implicit and explicit motor imagery tap different representations of the body.

The fact that ratings of Present pain, but not Pain with movement, correlate with performance on explicit motor imagery tasks whereas the opposite pattern is found with tasks that do not require explicit motor imagery [cf., (33)] is consistent with the view that Present pain ratings are mediated by the body image, a representation that incorporates conscious awareness of pain whereas ratings of Pain with movement are mediated by the body schema, a representation that is intimately linked to “forward models” of action [cf., (50)]. Multiple lines of evidence suggest that forward models provide information about the sensory consequences of action that it, in turn, feeds back to modify action as it unfolds (15, 51).

A second potential explanation concerns the nature of Present pain ratings as compared to Pain with action ratings. Normal nociception reflects a balance between inputs from small, unmyelinated fibers in response to specific, harmful stimuli, and central inhibition of these inputs. With sustained pain the balance between peripheral input and central inhibition may be altered; reduction of central pain inhibition may lead to “nociplastic” pain that is divorced from actual or threatened tissue damage (41–43, 52). It is possible that chronic back pain alters an individual's perception of their ability to perform any motor task. An explicit motor task requiring complex movements of the type queried in the Can They Touch task may be more susceptible to the effects of pain with action.

Finally, the absence of a direct assessment of the motor imagery abilities of participants is a weakness of the study. Although individual differences in motor imagery capacity may explain some of variability in RTs across subjects, we believe that it is not likely to be a major factor driving our results. Poor motor imagery would be expected to impact performance across all tasks and would not, for example, account for the significant effects of condition (e.g., upper body, lower body and cross body trials) or pain severity on performance.

## 5 Conclusions and future directions

We describe a novel task that provides a measure of low back pain severity that correlates with participant ratings of their pain severity. The effect is not simply attributable to the novelty of the task or non-specific factors such as medication effects or arousal, as the difference between asymptomatic and pain subjects was significant only for those items involving the lower back and legs but not the upper body. Furthermore, the difference between pain subjects and controls was greatest for subjects with severe back pain.

Future work should be undertaken to confirm the findings reported here in a larger sample of subjects with pain. Additionally, the potential utility of the Can they touch task as a marker for response to therapy (e.g., PT, surgery, medications, etc.) should be explored. We previously demonstrated in a small study (25, 26) of subjects with hand/shoulder pain that performance on a mental motor imagery task improved in tandem with subjects' ratings of their pain severity shortly after therapy. This finding raises the possibility that the Can they touch task may provide a dynamic index of pain severity that may provide a measure of treatment efficacy in one-time or longitudinal interventions.

## Data Availability

All data and stimuli are available online at https://osf.io/wv3xc/.

## Footnotes

^1^The same pattern of results was observed in comparisons of both pain groups to controls when analyzed independently.
